# Characteristics of 2 Different Commercially Available Implants with or without Nanotopography

**DOI:** 10.1155/2013/769768

**Published:** 2013-10-02

**Authors:** Ali Alenezi, Yoshihito Naito, Martin Andersson, Bruno R. Chrcanovic, Ann Wennerberg, Ryo Jimbo

**Affiliations:** ^1^Department of Prosthodontics, Faculty of Odontology, Malmö University, 205 06 Malmö, Sweden; ^2^Department of Oral and Maxillofacial Prosthodontics and Oral Implantology, Institute of Health Biosciences, The University of Tokushima Graduate School, 3-18-15 Kuramotocho, Tokushima 770-8504, Japan; ^3^Department of Chemical and Biological Engineering, Applied Surface Chemistry, Chalmers University of Technology, 412 96 Gothenburg, Sweden

## Abstract

The aim of this study was to assess histologically and histomorphometrically the early bone forming properties after 3 weeks for 2 commercially available implants, one supposedly possessing nanotopography and one without, in a rabbit femur model. Twenty-four implants divided equally into 2 groups were utilized in this study. The first group (P-I MICRO+NANO) was a titanium oxide (TiO_2_) microblasted and noble gas ion bombarded surface while the second group (Ospol) was anodic oxidized surface with calcium and phosphate incorporation. The implants were placed in the rabbit femur unicortically and were allowed to heal for 3 weeks. After euthanasia, the samples were subjected to histologic sectioning and bone-implant contact and bone area were evaluated histomorphometrically under an optical microscope. The histomorphometric evaluation presented that the P-I MICRO+NANO implants demonstrated significantly higher new bone formation as compared to the Ospol implants. Within the limitations of this study, the results suggested that nanostructures presented significantly higher bone formation after 3 weeks *in vivo*, and the effect of chemistry was limited, which is indicative that nanotopography is effective at early healing periods.

## 1. Introduction

Replacing a missing tooth with endosseous implants has been recognized as a long-term successful treatment option [1, 2]. Recent research trends further focus on enhancing the bone in apposition to the implant to ensure rapid and firm osseointegration. The major factors influencing the bone response around implants are reported to be the implant macrodesign, surface topography, and surface chemistry, which have been investigated in numerous studies [3–5]. Surface topography in particular has drawn significant attention as an important factor since it has been suggested that moderately rough implant surfaces present the strongest bone responses [6–8]. Alteration of the surface topography can be conducted in various methods, which provide unique characteristics [9]. Roughening the surface with blasting particles along with different types of acid etching is a commonly utilized technique to modify the surface topography [10, 11]. This method is unique in a way that the roughness can be altered in a controlled manner by changing the velocity, particle size, and particle properties [12]. Furthermore, the acid etching not only cleans off the remnants of the particles, but creates a unique surface topography, which has also been reported to alter the surface chemistry [13, 14]. It has been presented in numerous *in vivo* studies that this type of modification can enhance bone regeneration which is believed to be due increase in the surface area gained by surface roughness [10, 15–17]. With regards to the effect of chemistry, it has been reported that elements such as calcium and phosphate have significant influence on bone formation [18, 19]. It has also been reported that chemically modified surfaces provide a specific bonding between the implant and the surface, which has been described as biochemical bonding [20, 21]. One of the recognized methods to chemically modify the surface is the anodic oxidation technique. It is a method to increase the thickness of the oxide layer with a possibility to incorporate elements such as magnesium and phosphates and also provide a unique porous topography [20–23]. It has been proven that this modification significantly improves both the rate and quality of osseointegration [20].

In order to further enhance osseointegration, recent research has focused on modifying the topography at the nanolevel, since cells and proteins are reportedly interacting at this level [24, 25]. Reports suggest that *in vitro* these features could modify cellular shape and influence the migration and differentiation of mesenchymal stem cells [24–30]. It has been reported in several studies that the application of nanostructures increases the bioactivity of the implant surface which leads to an enhanced bone apposition around implants [31–33]. 

Although both nanotopographical and chemical modifications have proven to be an enhancing factor for osseointegration, it is of great interest to observe the bone forming characteristics of commercially available implants possessing either one of the factors. In this study, two commercially available implants, one supposedly possessing intended nanostructures formed by a noble gas ion bombardment and another chemically modified implant with calcium incorporated anodic oxidation, were characterized by various methods. Thereafter, the two commercially available implants were placed in the rabbit femur to observe histologically and histomorphometrically the early bone forming properties of 3 weeks.

## 2. Materials and Methods

### 2.1. Implant Surface Preparation and Characterization

Twenty-four implants divided into 2 groups were used in this study. The first group (*n* = 12) was a titanium oxide (TiO_2_) microblasted and noble gas ion bombarded surface (Functional hybrid implants, P-I MICRO+NANO, Zimmer Dental), with a diameter of 3.75 mm and length of 11.5 mm. 

 The second group (*n* = 12) was an anodic oxidized surface with calcium and phosphate incorporation (Ospol surface, Ospol, Zimmer Dental), with a diameter of 3.9 mm and length of 8.0 mm. 

### 2.2. Interferometer

Topographical analyses at microlevel were performed with interferometry (MicroXAM—PhaseShift, Tucson, AZ, USA). Following guidelines that were suggested by Wennerberg and Albrektsson [34], three implants from each group were examined in order to characterize the surface roughness. Each implant was examined at 9 different positions (3 top areas, 3 valley areas, and 3 flank areas). Parametric calculations were performed after errors of form and waviness were removed using a 50 × 50 mm Gaussian filter. Data was collected from the following three-dimensional parameters: the arithmetic mean of the height variation from a mean plane, Sa (*μ*m); the density of summits, Sds (*μ*m^−2^); and the developed surface area added by the roughness, Sdr (%). (Measurement area: 200 *μ*m × 250 *μ*m.) 

The evaluation was performed with the Surfascan software, and the images were produced using MounatinsMap universal 6.2 software.

### 2.3. Atomic Force Microscopy

The topographies of P-I MICRO+NANO and Ospol surfaces were characterized at nanolevel using atomic force microscopy (XE-100, Park systems). The analysis was performed in noncontact mode using silicon nitride probe with a nominal resonance frequency between 200 and 400 kHz (ACTA-10, APPNANO, USA). For this test, discs with the same implant surface treatment were used (3 discs for each group). Measurements areas of (10 × 10) and (1 × 1) in three random positions were selected for each disc. The measurements were performed at a scan rate of 0.50 Hz. 

The raw data obtained from the topographical equipment were further processed to separate the form, waviness, and roughness from the original measurements. A Gaussian high, pass filter was used (25% of surface area). The Gaussian filter is suitable for smoothing surfaces with rich features. The parameters used to calculate surface roughness were the same ones used with interferometer which are: Sa, Sds, and Sdr. Analysis and processing of the AFM images were performed with the MountiansMap Universal 6.2 software.

### 2.4. Scanning Electron Microscopy

The surface morphologies of 2 discs from each group were examined by scanning electron microscopy (SEM) using an LEO Ultra 55 FEG (Zeiss, Oberkochen, Germany) at an accelerating voltage of 5 kV. A secondary electron in-lens detector was used for visualization.

### 2.5. X-Ray Photoelectron Spectroscopy

The surface chemistry was investigated using X-ray photoelectron spectroscopy (XPS) utilizing a Kratos Axis Ultra XPS instrument equipped with a monochromatic Al K*α* X-ray source. Binding energies between 0 and 1100 eV were monitored, 0.800 eV/step and 50 ms/step, at a pass energy of 187.85 eV (150 W). One implant from each group was examined.

### 2.6. Animals and Surgery

The study was approved by the Malmö/Lund, Sweden, Regional Animal Ethical Committee. Twelve rabbits were included of mixed sexes with an average weight of approximately 4 kg.

Before surgery, the animals were sedated by intramuscular injections of a mixture of 0.15 mL/kg of medetomidine (1 mg/mL Dormitor—Orion Pharma, Sollentuna, Sweden) and 0.35 mL/kg of ketamine hydrochloride (50 mg/mL Ketalar—Pfizer AB, Sollentuna, Sweden). The hind legs were shaved and disinfected with 70% ethanol and 70% chlorhexidine. Lidocaine hydrochloride (Xylocaine—AstraZeneca AB, Gothenburg, Sweden) was administrated as local anesthetic at each insertion site at a dose of 1 mL. After osteotomy preparation following the manufacturers instructions, the implants were inserted in both sides of the femur. After the operation, buprenorphine hydrochloride (0.5 mL Temgesic—Reckitt Benckiser, Slough, UK) was administered as an analgesic for 3 days. After 3 weeks, the rabbits were euthanized with an overdose (60 mg/mL) of pentobarbital natrium (Apoteksbolaget AB, Stockholm, Sweden).

### 2.7. Histology and Histomorphometry

After euthanasia, the samples were processed in series of dehydrations in ethanol and infiltrations in resin; they were embedded in light-curing resin (Technovit 7200 VLC—Heraeus Kulzer, Wehrheim, Germany). Thereafter, the resin-embedded samples were subjected to undecalcified ground sectioning. One central ground section was prepared from each block by using the Exakt sawing and grinding equipment [35]. The sections were ground to a final thickness of approximately 20 *μ*m and histologically stained with toluidine blue and pyronin G.

Histological evaluations were performed using a light microscope (Eclipse ME600—Nikon Co., Tokyo, Japan), and the histomorphometrical data were analyzed by image analysis software (Image J v. 1.43u—National Institutes of Health, Bethesda, MD, USA). The bone-implant contact (BIC) percentage and the bone area (BA) and the new bone area (new-BA) percentages along the implant for total bone and new bone were calculated. New bone formation surrounding the implants was used for evaluating the osteoconductivity.

### 2.8. Statistical Analysis

For the histological evaluation, the wilcoxon rank-sum test was used for statistical analysis. For interferometer and AFM measurements, the mean values of surface roughness were compared with those of Independent-samples *t*-test using Statistical Package for the Social Sciences (SPSS) version 20 software (SPSS Inc., Chicago, USA). The degree of statistical significance was considered *P* < 0.05.

## 3. Results

### 3.1. Topographical Characterization

The results of the interferometer measurements are presented in Table 1. The average height deviation (Sa) was significantly different between the two groups (*P* = 0.000). Both surfaces were smooth according to the definition by Albrektsson and Wennerberg [3].

Further, the number of summits per unit area (Sds) differed significantly among the surfaces, with the Ospol surface presenting higher values than the P-I MICRO+NANO surface (*P* = 0.000). 

**Figure 1:**
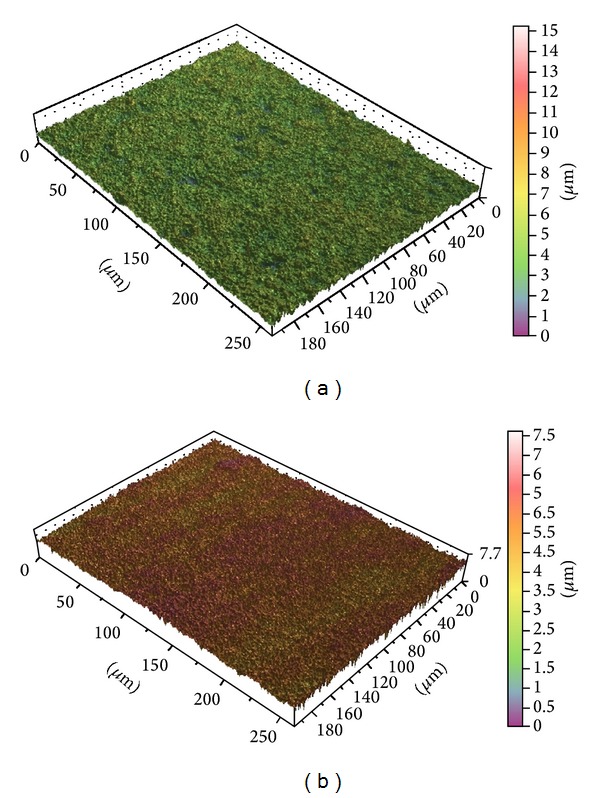
Representative interferometer images of 3D surface topography of the (a) P-I MICRO+NANO and (b) Ospol discs.

The surface enlargement percentage (Sdr) showed that the Ospol surface had a significantly larger surface area than the P-I MICRO+NANO surface (*P* = 0.028).  Figure 1 shows Interferometer images of 3D surface topography of the P-I MICRO+NANO and Ospol discs.

In contrast to the interferometer analysis, high-resolution topographical analysis with the AFM showed decreased surface roughness for the P-I MICRO+NANO compared to Ospol discs. The Sa, Sds, and Sdr parameters of the two groups at 10 × 10 scan size are presented in Table 2. The statistical analysis showed significant differences in Sdr and Sds values (*P* = 0.027 and 0.002, resp.).

**Figure 2:**
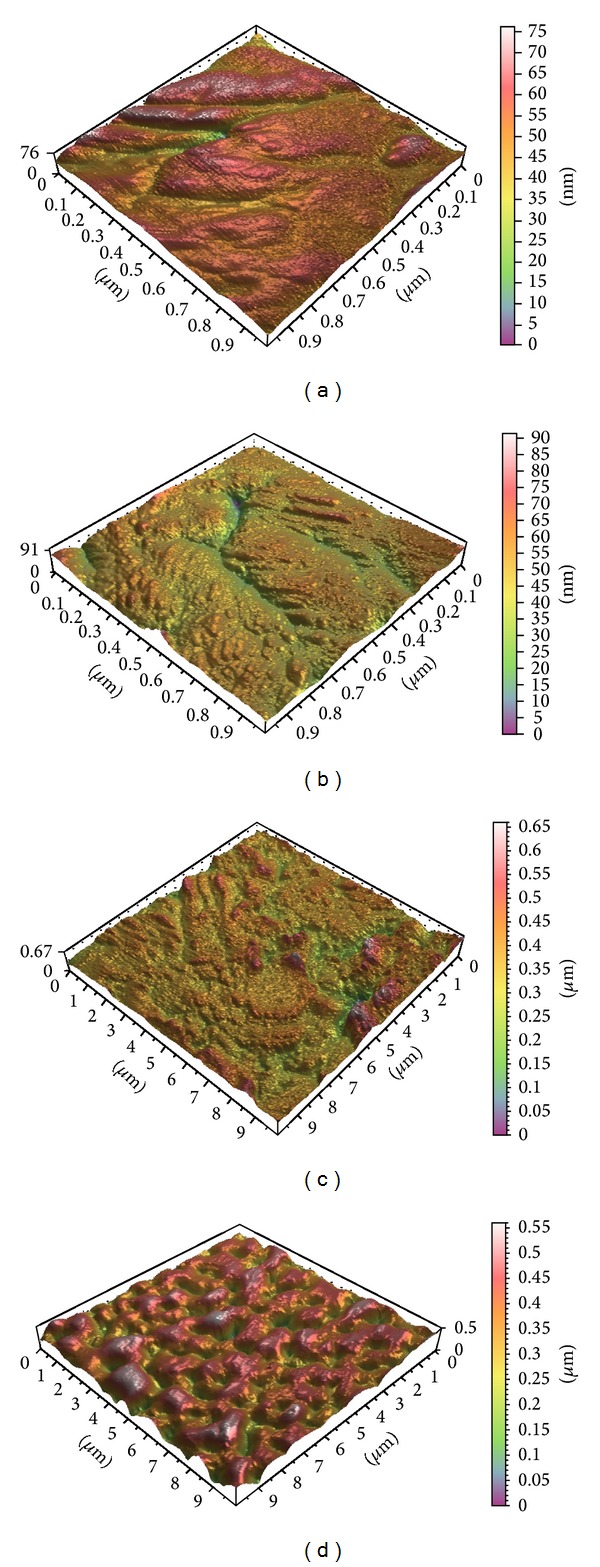
Representative AFM images of the 3D surface topography of (a) P-I MICRO+NANO, (b) Ospol discs at 1 × 1 scan area, (c) P-I MICRO+NANO, and (d) Ospol discs at 10 × 10 scan area.

For the 1 × 1 scan size, the Sa, Sds, and Sdr parameters are presented in Table 3. The statistical analysis shows significant difference only in the Sds value (*P* = 0.035). Representative AFM images of the 3D surface topography of the P-I MICRO+NANO and Ospol discs for both scan ranges are presented in Figure 2.

### 3.2. Scanning Electron Microscopy

**Figure 3:**
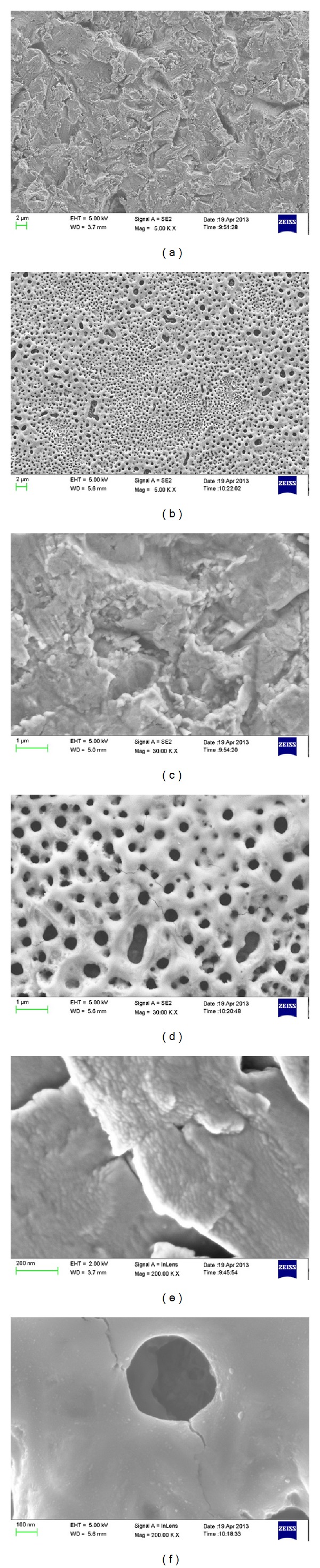
SEM images for the discs surfaces show (a) P-I MICRO+NANO and (b) Ospol at 5Kx magnification, (c) P-I MICRO+NANO and (d) Ospol at 30Kx magnification and (e) P-I MICRO+NANO and (f) Ospol at 200Kx magnification.

Scanning electron microscopy images of the P-I MICRO+NANO and Ospol discs are presented in Figure 3. At high magnification, P-I MICRO+NANO surface showed distinct, distributed, nanosized bumps with nanoparticles less than 100 nm in size. Ospol surface on the other hand showed extremely smooth surface with porous structures distributed on the surface with a diameter of approximately 300–500 nm.

### 3.3. X-Ray Photoelectron Spectroscopy

**Figure 4:**
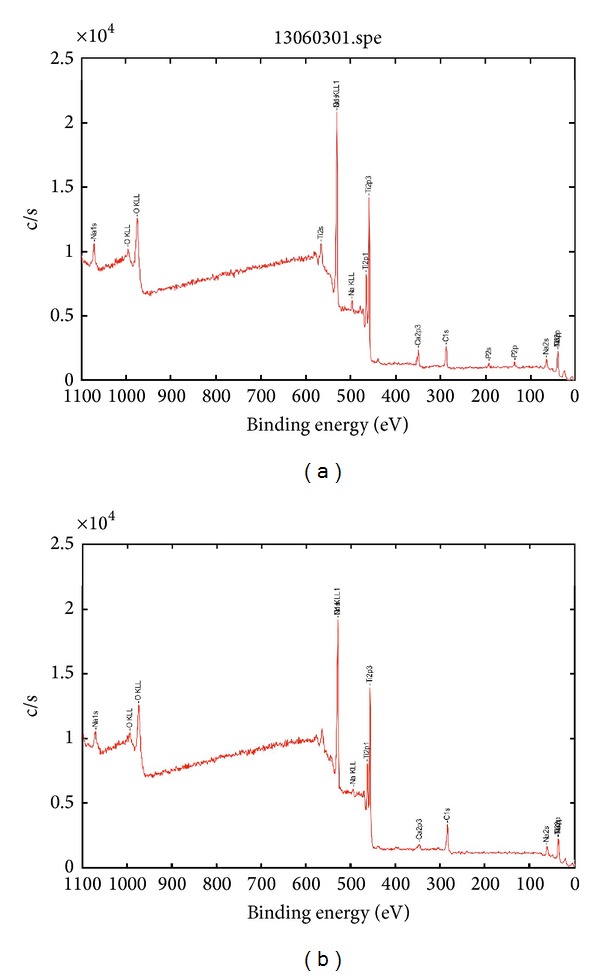
XPS survey spectra (a) Ospol and (b) P-I MICRO+NANO implants.

XPS survey spectra for the P-I MICRO+NANO and Ospol implants are presented in Figure 4, and the surface chemical composition is presented in Table 4. As it can be seen in the table, the largest difference between the two surfaces is the presence of calcium and phosphate on the Ospol surface. However, a relatively small amount of calcium was observed also on the P-I MICRO+NANO surface. Carbon and small quantities of nitrogen were present on both implants, which most probably is due to contamination.

### 3.4. Histology and Histomorphometry

**Figure 5:**
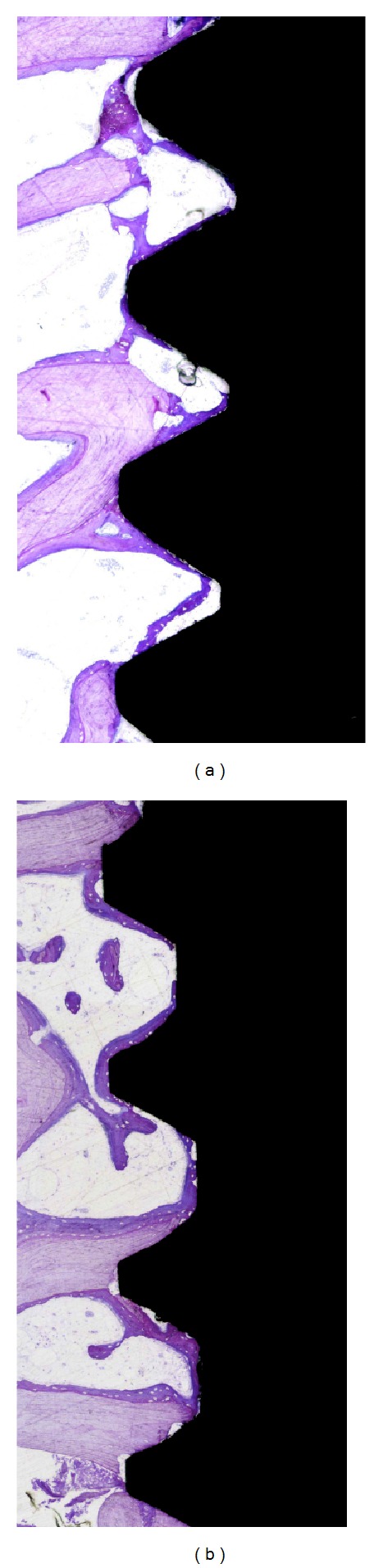
Histological observations of P-IMICRO+NANO implant (a) and Ospol implant (b) after 3 weeks (toluidine blue and pyronin staining, original magnification ×10). Cortical old bone is visualized in pale red while the New Bone is visualized in dark red.

A descriptive histologic image for both groups is presented in Figure 5. In brief, deeply stained woven bone was formed along the implant, which was in close contact for both groups. No signs of inflammation or bone resorption were evident.

The mean BIC values for all implant threads and for the top 3 threads of P-I MICRO+NANO and Ospol implants demonstrated no significant differences (*P* = 0.906, *P* = 0.87, resp.). When measuring the osteoconductivity of the surfaces, the two groups did not differ in BA% for both all threads and top 3 threads (*P* = 0.624, *P* = 0.583, resp.). 

**Figure 6:**
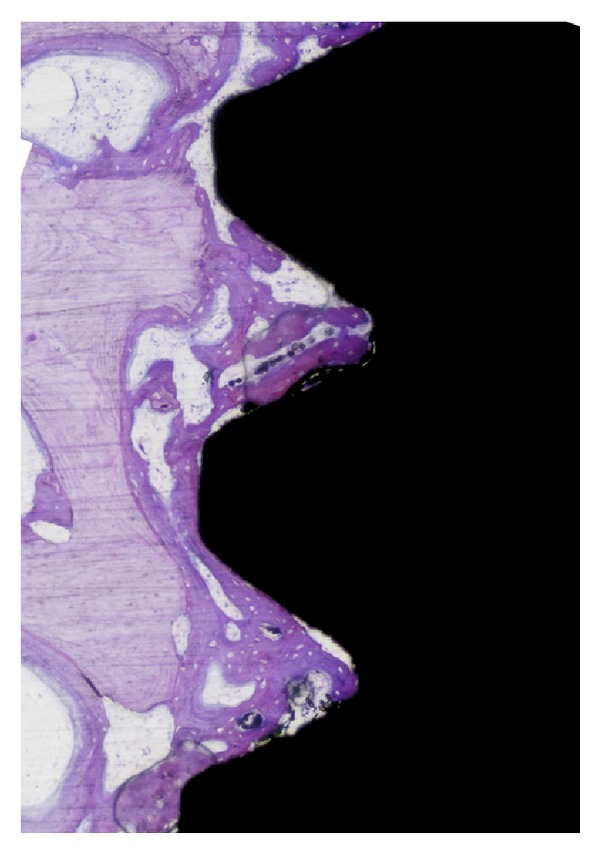
Histological observations of new bone formation among the threads of P-I MICRO+NANO implant after 3 weeks (toluidine blue and pyronin staining, original magnification ×10). Cortical old bone is visualized in pale red while the New Bone is visualized in dark red.

The new bone formation presented that the P-I MICRO+NANO implants which had significantly higher new bone are between all threads and the top 3 threads (*P* = 0.034, *P* = 0.025, resp.).  Table 5 summarizes all histomorphometric measurements.  Figure 6 shows a descriptive histological image for new bone formation in P-I MICRO+NANO implants after 3 weeks.

## 4. Discussion

 In the present study, 2 commercially available implants with and without nanostructures were chemically and topographically characterized, and the bone forming properties were evaluated after 3 weeks *in vivo* in a rabbit femur model. 

 From the SEM observations, the Ospol surface presented an extremely smooth morphology, and nanostructures in size of 100 nm or less could not be seen. The anodic oxidation process of the surface showed a typical surface morphology with porous structures of 300–500 nm in size. On the other hand, the SEM images for the P-I MICRO+NANO surface presented a typical surface morphology as a result of the TiO_2_ particle blasting with homogeneous nanostructures of about 20 nm in diameter.

The surface topography in the microlevel confirmed by the interferometer presented unique differences for both surfaces. It was confirmed that both surfaces were smooth according to the report from Wennerberg and Albrektsson [36]. The average height deviation (Sa) was significantly higher for the P-I MICRO+NANO surface, probably due to the topography created by the surface roughening procedure. However, the density of summits (Sds) and surface enlargement ratio (Sdr) were significantly higher for the Ospol, probably due to the existence of porous structures. The evaluation in the nanolevel confirmed by the AFM presented that the Sds was significantly higher for the P-I MICRO+NANO, which is an indication that the surface has been roughened in the nanoscale. The investigation of the chemical composition of the 2 different surfaces presented that the P-I MICRO+NANO surface presented a high amount of TiO_2_ due to the surface blasting procedure and the Ospol surface had large amounts of Ca and P on its surface, probably due to the anodic oxidation procedure performed in baths with these elements. 

 The histomorphometric measurements presented no significant differences in BIC or in total BA. However, when analyzing the amount of new bone formation represented by deeply stained tissue, significantly higher percentage in favor of the P-I MICRO+NANO implant was shown. The results strongly suggest that both surfaces have abundant osseointegration properties. This was evident in the representative histologic micrographs, where newly formed bone extended from the trabecular bone almost encapsulated the implant surface. The fact that the P-I MICRO+NANO surface presented higher new bone formation within the implant chamber is an indication that the slightly, but significantly, higher microtopography and the presence of the homogeneous nanotopography had positive effects on the bone. Although the Ospol surface underwent a chemical modification incorporating Ca and P into the surface, the histomorphometric results indicated that the chemical modification did not seem to have a strong influence on bone regeneration and the effect of topography was more significant. It is difficult to draw conclusions whether the topography or the chemistry plays a decisive role on bone formation, since some studies suggest the effect of nanotopography to be an influential factor [37, 38] and some others suggest that the effect of chemistry is of most importance [39, 40]. However, in cases where the nanostructure itself is consisting from CaP, it seems that there is a synergistic effect, with the bone mineralization properties being significantly enhanced [32, 41, 42]. It can be said that both chemical and topographical modifications are of great importance for osseointegration; however, their biologic effects may be dependent on numerous factors. More studies are necessary to determine the optimal surface that would present the strongest bone responses, and longer time points are warranted to observe the effect during longer healing periods.

## 5. Conclusion

The results suggested that the effect of homogenous nanostructures presented significantly higher bone formation after 3 weeks *in vivo*, which suggests the effect of nanotopography at early healing periods.

## Figures and Tables

**Table 1:** Surface roughness measurements for implants of the two groups using interferometer (9 measurements/implant, *n* = 3).

Sample	Sa *µ*m (SD)	Sds/mm² (SD)	Sdr % (SD)
P-I MICRO+NANO	0.6070 *µ*m (0.075)	186758 mm² (20462)	30.39% (8.45)
OSPOL	0.3599 *µ*m (0.090)	244573 mm² (41090)	44.08% (32.25)

**Table 2:** Surface roughness measurement using the AFM (scan size 10 × 10).

Scan size 10 × 10	Sa *µ*m (SD)	Sdr % (SD)	Sds 1/*µ*m^2^ (SD)
P-I MICRO+NANO	0.051 (0.007)	8.49 (3.59)	9.59555 (5.20)
OSPOL	0.058 (0.005)	11.44 (1.27)	3.08 (0.30)

**Table 3:** Surface roughness measurement using the AFM (scan size 1 × 1).

Scan size 1 × 1	Sa nm (SD)	Sdr % (SD)	Sds 1/*µ*m^2^ (SD)
P-I MICRO+NANO	4.35 (1.36)	8.56 (7)	1533 (671)
OSPOL	5.18 (1.11)	8.9 (3.70)	2295.2 (734)

**Table 4:** Surface chemical composition (atomic %) using XPS.

Element	C1s	N1s	O1s	P2p	Ca2p	Ti2p
P-I MICRO+NANO	22.75	1.07	56.77		0.88	18.53
OSPOL	17.51	0.46	58.88	2.38	2.23	18.55

**Table 5:** Summary of the histomorphometric measurements.

Group	BIC %	BIC %	BA %	BA %	New BA %	New BA %
	(All threads) (SD)	(Top 3 threads) (SD)	(All threads) (SD)	(Top 3 threads) (SD)	(All threads) (SD)	(Top 3 threads) (SD)
P-I MICRO+NANO	54.33 (14.7)	59.25 (12.9)	54.75 (8.4)	57.166 (10.1)	46.416 (9.4)	47.25 (10.15)
OSPOL	52.75 (15)	58.08 (14.7)	52.41 (13.8)	59.083 (13.9)	35.83 (10.6)	36.416 (11.3)
